# Telocytes in minor salivary glands of primary Sjögren’s syndrome: association with the extent of inflammation and ectopic lymphoid neogenesis

**DOI:** 10.1111/jcmm.12545

**Published:** 2015-03-06

**Authors:** Alessia Alunno, Lidia Ibba-Manneschi, Onelia Bistoni, Irene Rosa, Sara Caterbi, Roberto Gerli, Mirko Manetti

**Affiliations:** a Rheumatology Unit, Department of Medicine, University of PerugiaPerugia, Italy; b Department of Experimental and Clinical Medicine, Section of Anatomy and Histology, University of FlorenceFlorence, Italy

**Keywords:** primary Sjögren’s syndrome, focal lymphocytic sialadenitis, minor salivary glands, stromal cells, telocytes, immunohistochemistry

## Abstract

It has been recently reported that telocytes, a stromal (interstitial) cell subset involved in the control of local tissue homeostasis, are hampered in the target organs of inflammatory/autoimmune disorders. Since no data concerning telocytes in minor salivary glands (MSGs) are currently available, aim of the study was to evaluate telocyte distribution in MSGs with normal architecture, non-specific chronic sialadenitis (NSCS) and primary Sjögren’s syndrome (pSS)-focal lymphocytic sialadenitis. Twelve patients with pSS and 16 sicca non-pSS subjects were enrolled in the study. MSGs were evaluated by haematoxylin and eosin staining and immunofluorescence for CD3/CD20 and CD21 to assess focus score, Tarpley biopsy score, T/B cell segregation and germinal center (GC)-like structures. Telocytes were identified by immunoperoxidase-based immunohistochemistry for CD34 and CD34/platelet-derived growth factor receptor α double immunofluorescence. Telocytes were numerous in the stromal compartment of normal MSGs, where their long cytoplasmic processes surrounded vessels and encircled both the excretory ducts and the secretory units. In NSCS, despite the presence of a certain degree of inflammation, telocytes were normally represented. Conversely, telocytes were markedly reduced in MSGs from pSS patients compared to normal and NSCS MSGs. Such a decrease was associated with both worsening of glandular inflammation and progression of ectopic lymphoid neogenesis, periductal telocytes being reduced in the presence of smaller inflammatory foci and completely absent in the presence of GC-like structures. Our findings suggest that a loss of MSG telocytes might have important pathophysiological implications in pSS. The specific pro-inflammatory cytokine milieu of pSS MSGs might be one of the causes of telocyte loss.

## Introduction

Primary Sjögren’s syndrome (pSS) is a systemic autoimmune disorder mainly affecting women during the fourth and fifth decades of life (9:1 female to male ratio) and characterized by chronic inflammation of exocrine glands leading to progressive functional impairment 1–3. The histological hallmark of pSS is a focal lymphocytic sialadenitis (FLS) and the presence of at least one focus (*i.e*. an aggregate of at least 50 lymphocytes and plasma cells) in 4 mm^2^ of minor salivary gland (MSG) tissue allows the diagnosis of pSS 4. It is now well-established that the development and perpetuation of chronic inflammation in pSS and other disorders is the consequence of a complex interaction between immune cells and non-immune, tissue-resident, stromal (interstitial) cells. Indeed, stromal cells are no longer believed to be ‘innocent bystanders’ as a growing number of data suggests that they are active players in the induction and maintenance of the inflammatory process 5,6. For example, stromal cells isolated from an inflammatory microenvironment, such as rheumatoid synovium, drive the migration of immune cells *in vitro* 7. In addition, synovial- and MSG-resident stromal cells are able to secrete a variety of soluble mediators, including cytokines and chemokines that allow the transformation of diffuse infiltrates into highly organized structures. This process, called ectopic lymphoid neogenesis, culminates in the formation of tertiary lymphoid structures, namely germinal center (GC)-like structures 8–10.

Stromal cells include fibroblasts, dendritic cells, vascular endothelial cells and pericytes, but an additional type of stromal cells with peculiar phenotypical features has been recently identified and characterized in human and animal tissues 11–16. These cells, named telocytes (telos, *i.e*. provided with long-distance cell projections), display a small cell body and extremely long cytoplasmic processes (telopodes) with a moniliform aspect characterized by the combination of thin segments (podomers) and dilated regions (podoms) 13,15,16. The peculiar ultrastructural phenotype is currently considered as the most reliable hallmark for these cells, which do not possess unique immunophenotypic characteristics 13,15. However, at present, CD34 and platelet-derived growth factor receptor α (PDGFRα) are the best available markers for the immunohistochemical identification of telocytes under light microscopy 13–15,17.

According to their location in different organs and tissues, telocytes have been proposed to participate in a wide range of physiological processes such as the maintenance of local tissue homeostasis, tissue regeneration and intercellular signalling, and may exert their effects either *via* intercellular connections or *via* the release of extracellular vesicles and exosomes and the secretion of soluble mediators such as interleukin-6, VEGF and nitric oxide 12–15,18–21. As recently proposed, telocytes might even be considered as active players in immunomodulation and immune surveillance, acting like ‘local data suppliers’ for the immune response 15,19,20.

In recent years, telocytes have been investigated also in pathological processes, such as chronic inflammation, autoimmunity and fibrosis. Previous studies reported a marked reduction in telocytes in the skin and in other organs targeted by the fibrotic process including myocardium, lung and gastrointestinal tract of systemic sclerosis (SSc) patients 22,23. In addition, a disappearance of telocytes was observed in intestinal wall biopsies of patients with inflammatory bowel diseases, including Crohn’s disease (CD) and ulcerative colitis (UC) 24,25. According to the aforementioned evidence, it is conceivable that telocytes might be involved in the pathophysiology of multiple autoimmune inflammatory disorders, similar to other stromal cells 5,6.

As far as SGs are concerned, to date the only study available described telocyte distribution in normal parotid glands reporting that these peculiar stromal cells surround secretory and excretory structures, namely acini and ducts, and are also in close contact with blood vessels 26.

Taken the lack of data in normal and pSS MSG tissue, the aims of the present study were to evaluate telocyte distribution in MSGs with normal architecture, non-specific chronic sialadenitis (NSCS; *i.e*. presence of scattered lymphocyte aggregates that do not reach the number of 50 and therefore cannot be classified as foci) and FLS 27 and to identify possible associations between telocyte patterns and the extent of glandular inflammation and lymphoid organization.

## Materials and methods

### Study population

Twelve female patients diagnosed with pSS referring to the Rheumatology Unit, University of Perugia, Italy, were retrospectively evaluated. All patients fulfilled the American-European consensus criteria for pSS, including histopathological criteria 28. The presence of concurrent autoimmune/inflammatory disorders was ruled out. None of the patients was taking immunosuppressive drugs or corticosteroids. Sixteen female subjects with sicca symptoms but without any clinical and serological features of pSS were also enrolled as controls. Of these, 8 subjects displayed normal MSGs and 8 displayed a certain degree of MSG inflammation (NSCS) but no evidence of FLS. The whole study was approved by the local Ethic Committee. Written informed consent was obtained from all subjects, in accordance with the declaration of Helsinki.

### Salivary gland specimens

All participants underwent labial MSG biopsy for diagnostic purposes and 5/6 lobules were blindly analyzed for each sample. MSG samples were fixed in formalin, dehydrated in graded alcohol series and embedded in paraffin. For routine histopathological analysis, MSG sections (3 μm thick) were deparaffinized, rehydrated and stained with haematoxylin and eosin. Focus score and Tarpley biopsy score (0–4 scale) 29 were performed on haematoxylin and eosin-stained sections.

### Immunofluorescence staining

Cellular infiltrate and lymphoid organization were assessed by immunofluorescence staining of serial sections with antibodies against human CD3 (T-cell marker), CD20 (B-cell marker) and CD21 (marker of follicular dendritic cells characteristic of GC-like structures). T/B cell segregation was assessed by CD3/CD20 double immunofluorescence staining. Telocytes were identified by CD34 and PDGFRα immunofluorescence, according to previously published protocols 17,23–25. Double immunofluorescence staining for CD34 and PDGFRα, CD31 (pan-endothelial cell marker) or CD21 was also performed. Paraffin-embedded MSG tissue sections (3 μm thick) were deparaffinized, rehydrated and boiled for 10 min. in sodium citrate buffer (10 mM, pH 6.0). Sections were washed in PBS, incubated in 2 mg/ml glycine for 10 min. to quench autofluorescence caused by free aldehydes, and then blocked for 1 hr at room temperature with 1% bovine serum albumin (BSA) in PBS. The sections were then incubated overnight at 4°C with the following primary antibodies diluted in PBS with 1% BSA: rabbit monoclonal anti-human CD3 (1:100 dilution; catalogue number ab109531; Abcam, Cambridge, UK), mouse monoclonal anti-human CD20 (1:200 dilution; clone L26, catalogue number M0755; Dako, Glostrup, Denmark), rabbit monoclonal anti-human CD21 (1:200 dilution; catalogue number ab75985; Abcam), mouse monoclonal anti-human CD34 (1:50 dilution; clone QBEnd-10, catalogue number M7165; Dako), goat polyclonal anti-human PDGFRα (1:100 dilution; catalogue number AF-307-NA; R&D Systems, Minneapolis, MN, USA) and rabbit polyclonal anti-human CD31/platelet-endothelial cell adhesion molecule-1 (PECAM-1; 1:50 dilution; catalogue number ab28364; Abcam). The day after, the slides were washed three times in PBS and incubated for 45 min. at room temperature in the dark with Alexa Fluor-488-conjugated goat antimouse IgG, Alexa Fluor-488-conjugated donkey antimouse IgG, Rhodamine Red-X-conjugated goat anti-rabbit IgG or Alexa Fluor-568-conjugated donkey anti-goat IgG (Invitrogen, San Diego, CA, USA) diluted 1:200 in PBS with 1% BSA, as secondary antibodies. Double immunofluorescence staining was performed by mixing mouse and rabbit or goat primary antibodies and subsequently mixing appropriate fluorochrome-conjugated secondary antibodies. Irrelevant isotype-matched and concentration-matched mouse, rabbit and goat IgG (Sigma-Aldrich, St. Louis, MO, USA) were used as negative controls. Cross-reactivity of secondary antibodies was tested in control experiments in which primary antibodies were omitted. Nuclei were counterstained with 4′,6-diamidino-2-phenylindole (DAPI; Chemicon International, Temecula, CA, USA). MSG sections were then mounted with an antifade aqueous mounting medium (Biomeda Gel Mount; Electron Microscopy Sciences, Foster City, CA, USA) and examined with a Leica DM4000 B microscope equipped with fully automated fluorescence axes (Leica Microsystems, Mannheim, Germany). Fluorescence images were captured with a Leica DFC310 FX 1.4-megapixel digital colour camera equipped with the Leica software application suite LAS V3.8 (Leica Microsystems).

### Immunoperoxidase-based immunohistochemistry

To further identify telocytes in MSG sections with an independent method, indirect immunoperoxidase-based immunohistochemistry was also performed. After deparaffinization and rehydration, MSG tissue sections (3 μm thick) were boiled for 10 min. in sodium citrate buffer (10 mM, pH 6.0) for antigen retrieval and treated with 3% H_2_O_2_ in methanol for 15 min. at room temperature to block endogenous peroxidase activity. Sections were then washed and incubated with Ultra V block (UltraVision Large Volume Detection System Anti-Polyvalent, HRP, catalogue number TP-125-HL; LabVision, Fremont, CA, USA) for 10 min. at room temperature according to the manufacturer’s protocol. After blocking non-specific site binding, slides were incubated overnight at 4°C with mouse monoclonal anti-human CD34 antibody (1:50 dilution; clone QBEnd-10, catalogue number M7165; Dako) diluted in 1% BSA in PBS. The day after, tissue sections were washed three times in PBS and incubated with biotinylated secondary antibodies (UltraVision Large Volume Detection System Anti-Polyvalent, HRP; Lab-Vision) for 10 min. at room temperature. Subsequently, the slides were washed three times in PBS and incubated with streptavidin peroxidase (UltraVision Large Volume Detection System Anti-Polyvalent, HRP; Lab-Vision) for 10 min. at room temperature. Immunoreactivity was developed using 3-amino-9-ethylcarbazole (AEC kit, catalogue number TA-125-SA; LabVision) as chromogen (brownish-red colour). MSG sections were finally counterstained with Mayer’s haematoxylin (Bio-Optica, Milan, Italy), washed, mounted in an aqueous mounting medium and observed under a Leica DM4000 B microscope equipped with fully automated transmitted light axes (Leica Microsystems). Sections not exposed to primary antibodies or incubated with isotype-matched and concentration-matched non-immune mouse IgG (Sigma-Aldrich) were included as negative controls for antibody specificity. Light microscopy images were captured with a Leica DFC310 FX 1.4-megapixel digital colour camera equipped with the Leica software application suite LAS V3.8 (Leica Microsystems).

## Results

### MSG histopathology

**Figure 1:**
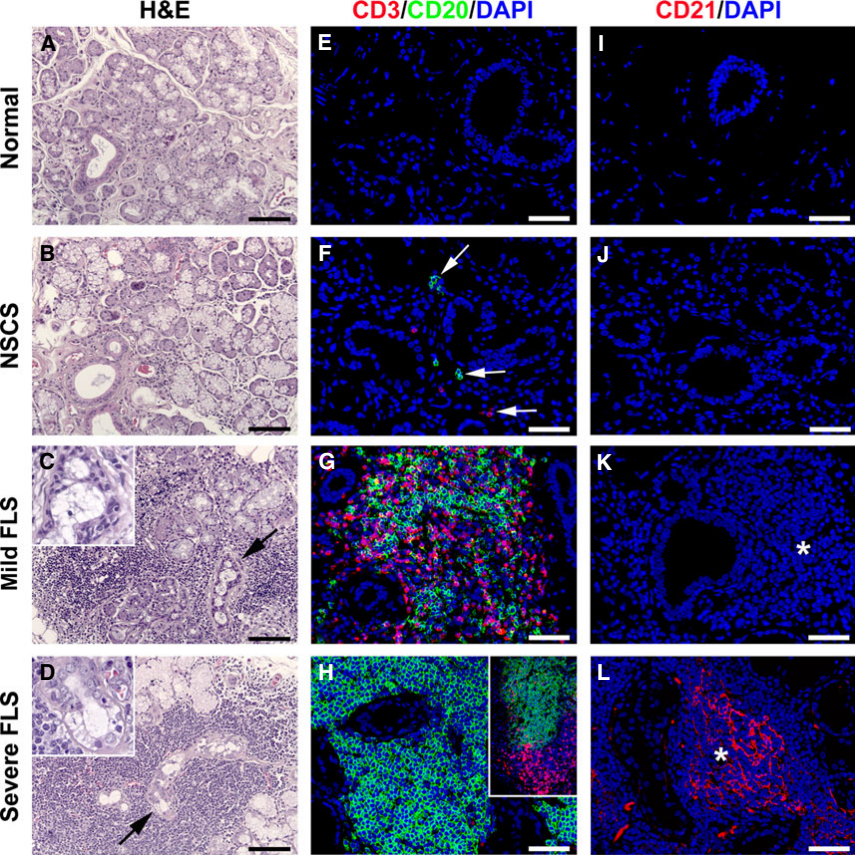
Histopathology and characterization of immune cell infiltration and lymphoid organization of minor salivary glands (MSGs). (A, E and I) Normal MSGs. (B, F and J) MSGs from non-specific chronic sialadenitis (NSCS). (C, G and K) MSGs from mild focal lymphocytic sialadenitis (FLS). (D, H and L) MSGs from severe FLS. (A–D) Haematoxylin and eosin staining. (C and D) Both mild FLS and severe FLS are characterized by the presence of periductal inflammatory aggregates replacing the secretory units. The extent of the inflammatory foci is greater in severe FLS than in mild FLS lesions. Higher magnification views of the duct regions pointed by arrows are shown in the insets. As compared with mild FLS, severe FLS displays reduced structural integrity of ducts with prominent epithelial cell apoptosis and loss (C and D, insets). (E–H) Double immunofluorescence staining for the T-cell marker CD3 (red) and the B-cell marker CD20 (green) with 4′,6-diamidino-2-phenylindole (DAPI, blue) counterstain for nuclei. (E) No lymphocytes are observed in normal MSGs. (F) Few scattered T and B cells are present in NSCS specimens (arrows). (G) T and B cells are interspersed within periductal inflammatory foci of mild FLS lesions. (H) B cells predominate in inflammatory foci of severe FLS lesions, with a clear segregation of T and B cells in different areas of the focus (inset). (I–L) Immunofluorescence staining for CD21 (red) with DAPI (blue) counterstain. (I and J) Both in normal and NSCS MSGs, no CD21^+^ cells are present. (K) In mild FLS, periductal inflammatory aggregates (asterisk) do not display germinal center (GC)-like structures. (L) In severe FLS, periductal inflammatory aggregates (asterisk) typically show GC-like structures identified by a network of CD21^+^ follicular dendritic cells. Original magnification: ×20 (A–D, H inset), ×40 (E–L), ×63 (C and D insets). Scale bar: 100 μm (A–D), 50 μm (E–L).

Findings of histopathological analysis of MSGs from patients with pSS and control cases are shown in Figure1A–L. On the basis of the severity of the histological lesions and the relative prevalence and segregation of T and B cells within the periductal inflammatory foci 30, MSG specimens from patients with pSS were categorized as mild FLS (*n* = 5) or severe FLS (*n* = 7). As compared with mild FLS, patients with severe FLS displayed more severe disorganization of the glandular architecture with larger periductal inflammatory aggregates replacing the secretory units and reduced structural integrity of ducts with prominent epithelial cell apoptosis and loss (Fig.1C, D and insets). This was reflected by the higher Tarpley biopsy score reported in severe FLS (score 4) than in mild FLS (score 1–3). As displayed in Figure1G, CD3/CD20 double immunofluorescence staining revealed that T and B cells were interspersed within periductal inflammatory foci of mild FLS lesions. Conversely, severe FLS lesions were characterized by B-cell predominance and a clear segregation of T and B cells in different areas within each focus (Fig.1H and inset). Moreover, while in severe FLS most of the periductal inflammatory aggregates displayed GC-like structures identified by CD21^+^ follicular dendritic cell network (Fig.1L), such structures were not detected in mild FLS (Fig.1K). As expected, no lymphocytes were found in normal MSGs (Fig.1E) and only few scattered T and B cells were observed in NSCS specimens (Fig.1F). Both in normal and NSCS MSGs, no CD21^+^ cells could be detected (Fig.1I and J).

### Distribution of telocytes in normal, NSCS and pSS MSGs

**Figure 2:**
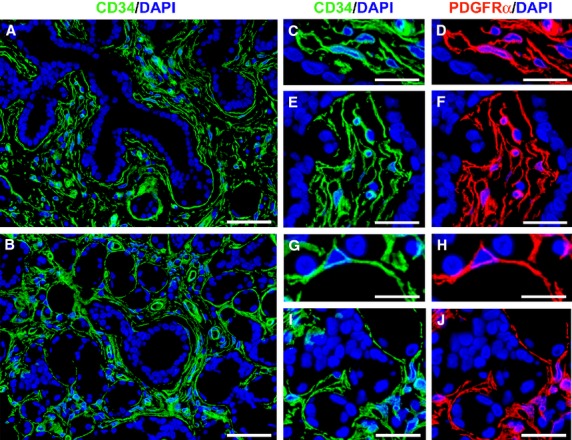
Distribution of telocytes in normal minor salivary glands. (A and B) Immunofluorescence staining for CD34 (green) with 4′,6-diamidino-2-phenylindole (DAPI, blue) counterstain for nuclei. (C–J) Double immunofluorescence staining for CD34 (green) and platelet*-*derived growth factor receptor α (PDGFRα, red) with DAPI (blue) counterstain. (A and B) Numerous CD34^+^ stromal cells surround vessels, excretory ducts and secretory units with their long cytoplasmic processes. (C–J) CD34^+^ stromal cells are also PDGFRα^+^ and show a slender nucleated body and long, thin and varicose processes, consistent with the diagnosis of telocytes. Scale bar: 50 μm (A and B), 20 μm (C–J).

**Figure 3:**
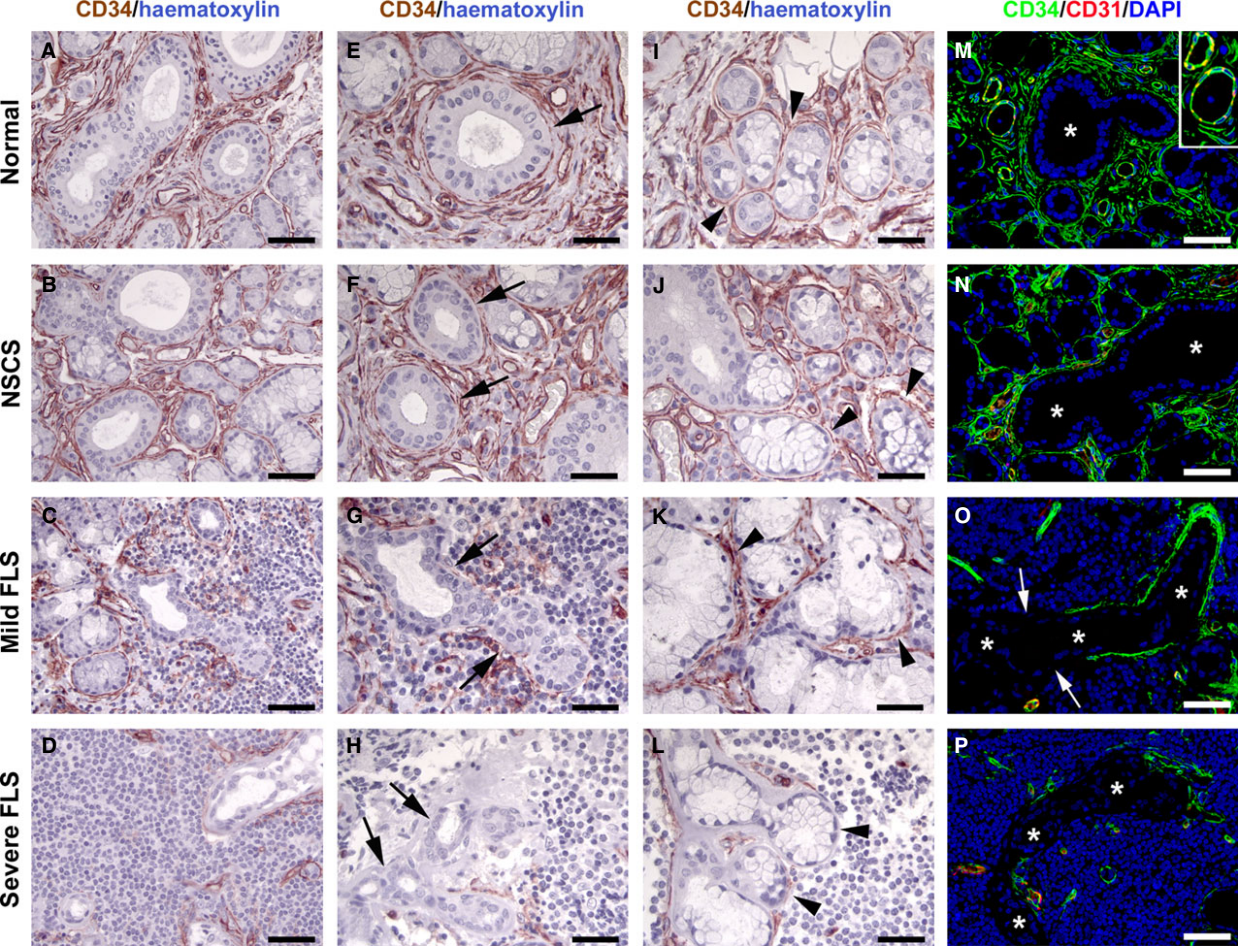
Differential distribution of telocytes in normal and diseased minor salivary glands (MSGs). (A, E, I and M) Normal MSGs. (B, F, J and N) MSGs from non-specific chronic sialadenitis (NSCS). (C, G, K and O) MSGs from mild focal lymphocytic sialadenitis (FLS). (D, H, L and P) MSGs from severe FLS. (A–L) CD34 immunoperoxidase staining (brownish-red) with haematoxylin counterstain. (A, E and I) Numerous telocytes can be observed in the stromal compartment of normal MSGs, where they surround vessels and form an almost continuous layer encircling both the ducts (E, arrow) and the acini (I, arrowheads). (B, F and J) In MSG sections from NSCS, telocyte distribution is comparable to that observed in normal MSGs, with numerous telocytes surrounding the ducts (F, arrows) and the acini (J, arrowheads). (C, G and K) In mild FLS lesions, a discontinuous layer of telocytes is present around the ducts involved by the inflammatory process (G, arrows), while telocyte distribution is preserved around the acini (K, arrowheads). (D, H and L) In severe FLS lesions, no telocytes can be observed around the ducts surrounded by the inflammatory foci (H, arrows), as well as around the acini involved by the inflammatory process (L, arrowheads). (M–P) Double immunofluorescence staining for CD34 (green) and the pan-endothelial cell marker CD31 (red) with 4′,6-diamidino-2-phenylindole (DAPI, blue) counterstain for nuclei. Both in normal MSGs (M) and NSCS MSGs (N), note the abundant network of CD34^+^/CD31^−^ telocytes surrounding vessels, acini and ducts (asterisks). The inset shows a higher magnification view of CD34^+^/CD31^+^ vessels surrounded by CD34^+^/CD31^−^ telocytes. (O) In mild FLS, a duct (asterisks) surrounded by inflammatory cells is only partially covered by telocytes. The ductal portion devoid of telocytes is pointed by arrows. (P) In severe FLS, a duct (asterisks) embedded in an inflammatory focus is completely devoid of telocytes. Original magnification: ×40 (A–D, M–P), ×63 (E–L). Scale bar: 50 μm (A–D, M–P), 30 μm (E–L).

According to previously published studies 14,17,22–25,31, telocytes were identified by CD34 and PDGFRα immunostaining, using both immunofluorescence and immunoperoxidase-based immunohistochemistry (Figs2 and 3).

Telocytes were numerous in the stromal compartment of normal MSGs, where their long cytoplasmic processes surrounded vessels and formed an almost continuous layer encircling both the excretory ducts and the secretory units (Figs2A and B, 3A, E and I). As displayed in Figure2C–J, these cells were CD34^+^/PDGFRα^+^ and, at higher magnification, showed a slender nucleated body and long, thin and varicose processes, the telopodes.

In MSG sections from NSCS cases, the distribution of telocytes was similar to that observed in normal MSGs (Fig.3B, F and J). Conversely, we detected abnormalities in telocyte distribution in all MSG biopsies from patients with pSS, with striking differences between mild and severe FLS cases. In mild FLS, telocytes were reduced resulting in a discontinuous layer around the ducts involved by the inflammatory process (Fig.3C and G). Instead, telocyte distribution appeared preserved around the acini (Fig.3K). In severe FLS, telocytes were almost completely absent around the excretory ducts surrounded by the inflammatory foci (Fig.3D and H). Moreover, telocytes also disappeared where the inflammatory infiltrate extended to surround the secretory units (Fig.3L).

The findings obtained using the immunoperoxidase-based methodology were confirmed by immunofluorescence analysis of MSG sections (Fig.3M–P). In addition, in agreement with previous reports 17,22–25, CD34/CD31 double immunofluorescence staining demonstrated that MSG telocytes were CD34^+^/CD31^−^, and therefore they could be clearly distinguished from CD34^+^/CD31^+^ vascular endothelial cells (Fig.3M–P and inset).

**Figure 4:**
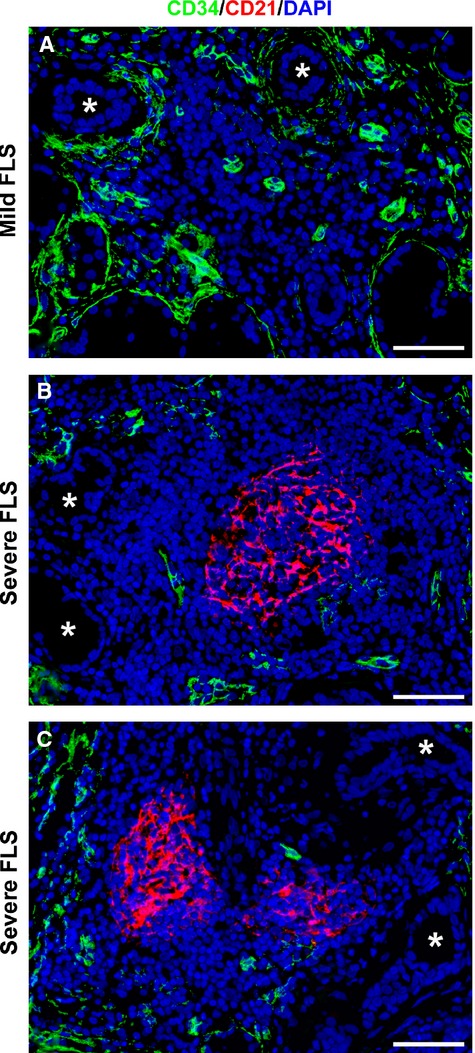
Differential distribution of telocytes according to the presence of germinal center (GC)-like structures in minor salivary glands (MSGs) from focal lymphocytic sialadenitis (FLS). (A–C) Double immunofluorescence staining for CD34 (green) and CD21 (red) with 4′,6-diamidino-2-phenylindole (DAPI, blue) counterstain for nuclei. Representative microphotographs of MSG sections from mild FLS (A) and severe FLS (B and C) are shown. Ducts are marked by asterisks. (A) Some telocytes are present around ducts surrounded by an inflammatory focus lacking GC-like structure. (B and C) Note the complete absence of telocytes around ducts surrounded by inflammatory foci displaying GC-like structures identified by CD21^+^ follicular dendritic cell network. Original magnification: ×40 (A–C). Scale bar: 50 μm (A–C).

To detect possible differences in telocyte distribution according to the presence of GC-like structures, we also performed CD34/CD21 double immunofluorescence on pSS MSG sections. Interestingly, no telocytes could be detected around ducts surrounded by inflammatory foci displaying GC-like structures, while some periductal telocytes were still present in the absence of GC-like structures (Fig.4A–C).

## Discussion

In the last decades, the pathogenic role of stromal cells in systemic inflammatory/autoimmune disorders including pSS has been extensively investigated 5,6. In this context, telocytes, a recently identified stromal cell subset involved in local tissue homeostasis, appear to be hampered in different conditions 22–25. Herein, we describe for the first time telocyte distribution in normal and inflamed MSGs. Unfortunately, we could not investigate the ultrastructural features of telocytes by transmission electron microscopy, as we had at our disposal paraffin-embedded MSG biopsies for diagnostic purposes which are suitable only for light microscopy. Nevertheless, in normal MSG tissue we could identify numerous CD34^+^/PDGFRα^+^ periacinar and periductal stromal cells displaying a slender nucleated body and long, thin and varicose processes consistent with the description of telocytes in the human gastrointestinal tract 17,24,25,32. Notably, telocytes might even correspond to the CD34^+^ dendritic interstitial cells previously described in SGs 33,34. In particular, we demonstrate that telocytes are markedly reduced in MSGs of patients with pSS with respect to normal and NSCS MSGs and such decrease parallels the worsening of inflammation and lymphoid organization.

A growing number of studies have described telocytes in different mammalian tissues and organs 11–21,35–40. Concerning SGs, the only study published to date was performed on rat and human normal parotid glands and revealed that telocytes are present not only in periacinar areas similarly to exocrine pancreas, but also around ductal structures as also observed in bile ducts 26,41. Our results in normal MSGs are in line with these observations, as telocytes appear to surround excretory ducts, secretory units and vessels. Therefore, since telocytes form a network interacting with different anatomical structures within MSGs and parotid glands, it is conceivable that they may participate in the maintenance of glandular homeostasis as proposed in other organs 15. The unique phenotype, ultrastructural characteristics, tissue distribution and multiple intercellular connections of telocytes, as well their putative role in local immune surveillance and homeostasis 15,19,20, raised the possibility of their involvement in the pathogenesis of inflammatory/autoimmune disorders. In this setting, numerical and structural abnormalities of this cell subset have been described in human CD, UC and SSc, as well as in experimental endometriosis 22–25,42. Taken that fibrosis following inflammation is a common hallmark in target tissues of these diseases, it has been speculated that telocyte entrapment in the fibrotic matrix may be one of the causes of their damage and progressive reduction in such scenarios 22–25. Furthermore, since telocytes may be more sensitive to ischemic injury compared to other stromal cells, a chronic ischemic microenvironment, as observed in SSc, may further contribute to their injury 22. More generally, in keeping with the proposed function as tissue homeostasis regulators, the local loss of telocytes might contribute to breaking out of immune homeostasis in the targeted organs of multiple autoimmune disorders.

As far as MSG inflammation is concerned, the evidence that telocytes are markedly reduced in FLS but not in NSCS deserves some consideration. In this setting, the formation and maintenance of FLS as well as the development of ectopic lymphoid structures during chronic inflammation are dependent on the expression of lymphotoxins, cytokines and chemokines by several cell types 8. We have previously reported that in NSCS MSGs the mRNA levels of several of these mediators are similar to that of normal MSGs, while they are significantly up-regulated in FLS compared to both normal and NSCS MSGs 43. These observations suggest that in pSS the peculiar inflammatory microenvironment might be the main responsible for local MSG telocyte damage and loss. Moreover, the fact that in pSS MSGs telocytes are preserved around the acini not affected by the inflammatory process may allow to speculate that the cross-talk between epithelial or infiltrating immune cells and telocytes occurs in a paracrine manner limited to each secretory unit. Indeed, it has been already demonstrated that telocytes may interact with other cells either *via* intercellular contacts or by the release of microvesicles, exosomes and paracrine mediators 12,13,15,16,18,19. In addition, while in CD lymphoid aggregates/granulomas are entirely surrounded by telocytes suggesting a certain attempt to control their spreading 24, the absence of telocytes around MSG inflammatory foci underscores that this mechanism may be impaired in pSS. Furthermore, the reduction in this cell subset appears to mirror the worsening of inflammation and the progression of ectopic lymphoid neogenesis in MSGs. Indeed, periductal telocytes were reduced in the presence of smaller inflammatory foci and completely absent in the presence of GC-like structures, thus reflecting disease severity. Notably, recent findings demonstrated that higher focus score and the presence of GC-like structures within MSGs may even predict the development of extraglandular manifestations including non-Hodgkin lymphoma in patients with pSS 43–45. In this setting, we previously demonstrated that pSS MSGs displaying GC-like structures show significantly higher levels of the aforementioned cytokines and chemokines compared to MSGs without GC-like structures 43.

In conclusion, our data demonstrate an overall impairment of telocyte network in MSGs during pSS. In line with the proposed functions of telocytes 13–15,22–25, it is conceivable that their loss might be involved in glandular architecture derangement and impaired homeostasis. However, whether the loss of telocytes in pSS represents either the cause or the consequence of local inflammation spreading remains still unknown. To further gain insights on the possible contribution of telocytes to pSS pathophysiology and the exact mechanisms underlying telocyte crosstalk with other MSG cell subsets and their disappearance during FLS, future ultrastructural and functional studies will be required.
